
JOURNAL INFORMATION
==============================
NLM Title Abbreviation: Biospektrum (Heidelb)
Journal ID: Biospektrum (Heidelb)
NLM Journal ID: 9615685

Biospektrum

ISSN: 0947-0867
EISSN: 1868-6249

ARTICLE INFORMATION
==============================
PMCID: PMC7318727
PMID: 32834540
DOI: 10.1007/s12268-020-1416-0
Article ID: 1416
Article version: 1
Subjects: Biotechnologie

Maßgeschneiderte Polyketidsynthasen zur Herstellung von Polyketid-Derivaten

Musiol-Kroll Ewa M. ewa.musiol@biotech.uni-tuebingen.de

Wohlleben Wolfgang
grid.10392.39 0000 0001 2190 1447 Interfakultäres Institut für Mikrobiologie und Infektionsmedizin (IMIT), Mikrobiologie/Biotechnologie Eberhard Karls Universität Tübingen, Auf der Morgenstelle 28, D-72076 Tübingen, Deutschland
Electronic publication date: 2020 Jun 26
Print publication date: 2020
Volume: 26
Issue: 4
First page: 437
Last page: 439
Received 2020 Jun 8; Accepted 2020 Jun 9
Copyright: © Die Autoren 2020
License: Open Access: Dieser Artikel wird unter der Creative Commons Namensnennung 4.0 International Lizenz veröffentlicht, welche die Nutzung, Vervielfältigung, Bearbeitung, Verbreitung und Wiedergabe in jeglichem Medium und Format erlaubt, sofern Sie den/die ursprünglichen Autor(en) und die Quelle ordnungsgemäß nennen, einen Link zur Creative Commons Lizenz beifügen und angeben, ob Änderungen vorgenommen wurden. Die in diesem Artikel enthaltenen Bilder und sonstiges Drittmaterial unterliegen ebenfalls der genannten Creative Commons Lizenz, sofern sich aus der Abbildungslegende nichts anderes ergibt. Sofern das betreffende Material nicht unter der genannten Creative Commons Lizenz steht und die betreffende Handlung nicht nach gesetzlichen Vorschriften erlaubt ist, ist für die oben aufgeführten Weiterverwendungen des Materials die Einwilligung des jeweiligen Rechteinhabers einzuholen. Weitere Details zur Lizenz entnehmen Sie bitte der Lizenzinformation auf http://creativecommons.org/licenses/by/4.0/deed.de.
License URL: https://creativecommons.org/licenses/by/4.0/

issue-copyright-statement: © Springer-Verlag GmbH Deutschland, ein Teil von Springer Nature 2020

==============================
Polyketides (PKs) are secondary metabolites with valuable properties, including antibiotic, antifungal, antiviral, anticancer, and immunosuppressive activity. The biosynthesis of PKs is accomplished by multifunctional megaenzymes, the polyketide synthases (PKSs). The molecular architecture of those remarkable assembly lines provides opportunities for their engineering and generation of PK derivatives with potentially improved pharmacokinetics and/or expanded spectrum of bioactivity.

■ Die steigende Anzahl von multiresistenten Keimen [1, 2] und die rasante Ausbreitung des Coronavirus SARS-CoV-2 [3] – es sind die aktuellen Ereignisse, die zum wiederholten Male die Notwendigkeit der Entwicklung von neuen Medikamenten deutlich machen.

Erythromycin, Epothilone, Vanitaracin A, Amphotericin B, Tacrolimus sind nur einige wenige Beispiele für Polyketide, die eine antibiotische, antitumorale, antivirale, fungizide bzw. immunsuppressive Wirkung zeigen. Polyketide bilden eine große Familie von Stoffen, die bezüglich ihrer Strukturen und chemischen sowie pharmakokinetischen Eigenschaften äußerst vielfältig sind. Dies macht die Polyketide nicht nur für den Einsatz in der Medizin attraktiv, sondern auch für die Landwirtschaft, Biotechnologie und viele andere Bereiche. Aufgrund der strukturellen Komplexität der Polyketide ist es jedoch oft nicht möglich, diese Klasse von Naturstoffen auf chemischem Wege herzustellen. Dagegen sind Mikroorganismen, vor allem Actinomyceten, in der Lage, eine Vielzahl dieser komplexen Sekundärmetaboliten zu produzieren. Dies ist hauptsächlich den Biosynthese-Genclustern (BGC), die sich im Genom der Produzentenstämme befinden, zu verdanken. Ein BGC beinhaltet typischerweise Gene, die für die Biosynthese, Resistenz und in den meisten Fällen auch für den Export der Polyketide codieren. Die Transkription und Translation der Biosynthesegene resultiert in der Bereitstellung von Schlüsselenzymen (Polyketidsynthasen, PKS) für die intrazelluläre Produktion der Polyketide [4]. Die PKS-Enzyme beruhen auf der Aktivität ihrer enzymatischen Domänen, welche die schrittweise Kondensation von Coenzym-A(CoA)-aktivierten Bausteinen katalysieren (**Abb. 1**). Dabei wird Acetyl-CoA oft als Startereinheit und Malonyl-CoA als Verlängerungseinheit verwendet. Eine „minimale PKS" besteht aus drei essenziellen Domänen: Acyltransferase (AT), Acyl-Carrier-Protein (ACP) und Ketosynthase (KS). Die Acyltransferase erkennt und bindet die CoA-aktivierten Substrate und überträgt die dabei entstandene Carbonsäure auf das ACP der PKS. Die Ketosynthase verknüpft zwei Vorstufen, beispielsweise eine Starter- mit einer Verlängerungseinheit oder die bereits synthetisierte Polyketidkette mit der neu geladenen Verlängerungseinheit. Des Weiteren können zusätzliche optionale Domänen, wie die Ketoreduktase (KR), Dehydratase (DH), Enoylreduktase (ER) und Methyltransferase (MT), Teil der PKS sein und die synthetisierte Polyketidkette modifizieren. Einige PKS-Enzyme sind mit einer Thioesterase-Domäne, die für die Termination und häufig auch Zyklisierung der Polyketidkette verantwortlich ist, ausgestattet. Die „PKS-Maschinerien" können sich in ihrer molekularen Organisation, Domänenzusammensetzung und der mechanistischen Art und Weise unterscheiden. Um die diversen Systeme auseinanderzuhalten, wurde eine Klassifizierung aufgestellt. Diese teilt die PKSs in drei Hauptklassen ein: Typ-I-PKSs (modulare Typ-I-PKSs und iterative Typ-I-PKSs) [5], Typ-II-PKSs (iterativ) [6] und Typ-III-PKSs (Chalkonsynthasen, iterativ) [7]. Obwohl die modularen Typ-I-PKS-Systeme eine enorme Komplexität aufweisen und somit eine Herausforderung für detaillierte Analysen und das *Engineering* darstellen, erlangten sie eine große Aufmerksamkeit der Wissenschaftler. Dies ist auch für unsere Arbeitsgruppe, die sich für die Biosynthese des Polyketid-Antibiotikums Kirromycin [8] interessiert, der Fall. Im Folgenden wird am Beispiel von Kirromycin aus *Streptomyces collinus* Tü365 nicht nur die Komplexität der modularen Typ-I-„Polyketid-Maschinerien", sondern auch das Potenzial der ATs für die Entwicklung von neuen Polyketid-Derivaten exemplarisch aufgezeigt.

## Komplexe Polyketid-Biosynthesewege

Die Besonderheit der Kirromycin-Biosynthese beruht auf der einzigartigen Kombination der biosynthetischen Schlüsselenzyme. Es handelt sich hier um die KirAI-KirAVI-Maschinerie, die aus fünf modularen Typ-I-PKSs und zwei nicht-ribosomalen Peptidsynthetasen (NRPSs) besteht (**Abb. 1**, [9]). NRPSs arbeiten nach einem ähnlichen Prinzip wie die modularen PKS-Enzyme: Aktivierte Aminosäuren (AS) werden auf die Carrierproteine der NRPS geladen und während der schrittweisen Biosynthese in die wachsende Polyketid- bzw. Peptidkette eingebaut.

In modular aufgebauten Typ-I-PKSs beinhaltet jedes Modul ein Set von enzymatischen Domänen, die für einen Zyklus der Polyketidkettenverlängerung benötigt werden. Diese sukzessive Polyketid-Biosynthese wird deswegen häufig auch als „Fertigungsstraße" oder „Fließband-Biosynthese"

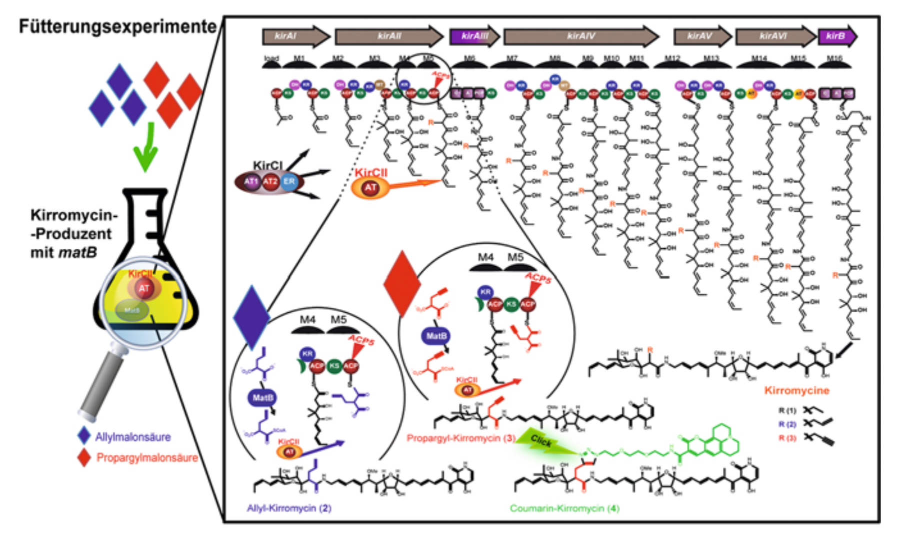

▲ **Abb. 1:** Kirromycin-Biosyntheseweg und KirCII-basierte Derivatisierung. Der modifizierte Kirromycin-Produzent exprimiert die Malonyl-CoA-Synthetase (T207G/M306I), die die Substrate Allylmalonsäure und Propargylmalonsäure aktiviert. Die dabei entstandenen Vorstufen (Allylmalonyl- und Propargylmalonyl-CoA) werden von der Acyltransferase KirCII auf das Acyl-Carrier-Protein im Modul M5 geladen und somit in die Polyketidkette eingebaut. Die Vervollständigung der Biosynthese führt zu der Entstehung von Kirromycin-Derivaten. A: Adenylierungsdomäne; AT: Acyltransferase; ACP: Acyl-Carrier-Protein; C: Kondensationsdomäne; DH: Dehydrogenase; ER: Enoylreduktase; KR: Ketoreduktase; KS: Ketosynthase; MatB: modifizierte Malonyl-CoA-Synthetase (T207G/M306I); MT: Methyltransferase; PCP: Peptidyl-Carrier-Protein; R: funktionelle Gruppe. KirCI und KirCII sind eigenständige Acyltransferasen.

bezeichnet. Dabei ist die essenzielle AT-Domäne in der Regel in die PKS-Enzyme „eingebettet“ (*cis*-AT-PKS). Die AT-losen PKSs (*trans*-AT-PKS) [10] stellen jedoch eine Ausnahme dar. Im Kirromycin-Biosyntheseweg wurden nicht nur NRPS-Module, sondern auch beide PKS-Subtypen im Laufe der Evolution miteinander kombiniert. KirAI-KirAV sind AT-los und werden somit dem *trans*-AT-PKS-Subtyp zugeordnet, KirAIV ist dagegen vom *cis*-AT-PKS-Subtyp (**Abb. 1**). Des Weiteren haben Genomsequenzanalysen ergeben, dass im Kirromycin-BGC zwei Gene, *kirCI* und *kirCII*, vorliegen, deren Genprodukte Ähnlichkeit zu Acyltransferasen zeigten. Demzufolge stellten sich die Fragen: Sind beide Enzyme (KirCI und KirCII) als eigenständige ATs aktiv? Mit welchen *trans*-AT-PKS interagieren sie, und welche Substratspezifität liegt vor?

## Eigenständige Acyltransferasen

Die Funktion und Substratspezifität von KirCI und KirCII wurden auf genetischer und biochemischer Ebene untersucht. Unsere Ergebnisse zeigten, dass KirCI und KiCII essenziell für eine effiziente Kirromycin-Produktion sind [11,12]. Beide Enzyme konnten in biochemischen Assays (ACP-Beladungsassays) als eigenständige ATs bestätigt werden. KirCI fungiert als Malonyl-CoA-spezifische AT und belädt mit hoher Wahrscheinlichkeit die ACPs der KirAI sowie die ersten drei Module der KirAII, KirAIV und KirAV mit Malonat (**Abb. 1**, [11]). Die Substratspezifität von KirCII hat sich als besonders interessant herausgestellt: KirCII agiert bei Standard-Kirromycin-Produktionsbedingungen als Ethylmalonyl-CoA-spezifische AT und transferiert Ethylmalonat nur auf ein einziges ACP des Kirromycin-Multienzymkomplexes, das ACP im Modul 5 (ACP5) (**Abb. 1**, [11]).

Weitergehende *in vitro*-Analysen des KirCII-Enzyms offenbarten, dass die AT in der Lage ist, zuzüglich zu Ethylmalonat auch alternative Vorstufen, wie Allyl- und Propargylmalonat und in geringerem Maße Phenyl- und Azidoethylmalonat, auf ACP5 zu transferieren. Darüber hinaus konnte der Einbau von Allyl- und Propargylmalonat in *in vivo*-Fütterungsstudien bestätigt werden. Die Experimente führten zur Isolierung von zwei Kirromycin-Derivaten, Allyl- und Propargyl-Kirromycin (**Abb. 1**, [13]). Letzteres besitzt eine Dreifachbindung (Alkingruppe), die sich besonders für Klick-Chemie-Reaktionen eignet und eine weitere Derivatisierung der Polyketidstruktur möglich macht. In Zusammenarbeit mit der Arbeitsgruppe von Prof. Dr. Stephanie Grond (Organische Chemie, Universität Tübingen) konnte die Klick-Chemie-Reaktion in Form einer Kupfer-katalysierten Alkin-Azid-Zykloaddition umgesetzt werden (**Abb. 1**). Ein weiteres Kirromycin-Derivat wurde hergestellt, dessen Struktur mittels NMR-Analyse als Coumarin-Kirromycin bestätigt wurde [13].

Die Ergebnisse dieser Studien demonstrieren die besondere Substrat- und ACP-Spezifität der KirCII-AT, die den „regiospezifischen“ Einbau von verzweigten Vorstufen in eine Polyketidstruktur ermöglicht. Diese Eigenschaften machen KirCII zum potenziellen Werkzeug für die Polyketid-Derivatisierung.

## Polyketid-Derivatisierung

Die strukturelle Diversität der Polyketide wird durch die Heterogenität der PKS-Systeme erreicht. Zu den Faktoren, die über die Struktur der Polyketid-Produkte entscheiden, gehören die molekulare Architektur der PKSs (Typ der PKS, Zusammensetzung der Module und Kombination der Domänen) und die Post-PKS-Modifikationsenzyme (*tailoring enzymes*). Relativ viele der strukturellen Variationen in Polyketiden sind jedoch auf die Substratspezifität der ATs und den verfügbaren Pool an Vorstufen zurückzuführen. In den vergangenen zwei Jahrzehnten wurde die strikte Korrelation der molekularen Architektur der modularen PKS und der Struktur der synthetisierten Polyketide (auch *co-linearity principle* genannt) verwendet, um die PKS zu manipulieren und so neue Polyketid-Derivate herzustellen. Zu den bekanntesten PKS-*Engineering*-Strategien gehören die Deletion, Insertion und der Austausch von kompletten Modulen oder einzelnen Domänen (*mix and match* oder *plug and play*) (**Abb. 2**). Die meisten dieser Modifikationen wurden für die PKSs aus dem Erythromycin-Biosyntheseweg erfolgreich durchgeführt. Gleichzeitig sind einige dieser Ansätze für weniger erforschte Polyketid-Biosynthesewege gescheitert. Offensichtlich sind die modularen PKS-Systeme komplexer als ursprünglich gedacht und müssen noch genauer unter die Lupe genommen werden. Neuere Studien der Ko-Evolution der gigantischen modularen PKSs offenbarten, dass die optionalen Domänen (prozessierende Domänen) eher mit der in der PKS stromabwärts vorliegenden Domäne als mit der stromaufwärts liegenden Domäne verwandt sind [14]. Diese Erkenntnis provozierte die Hypothese, dass ein Modul nicht wie bislang behauptet die Sequenz von der KS bis zur ACP (KS + AT + [DH + ER + KR] + ACP) hat, sondern mit einer AT „beginnt" und einer KS „endet" (AT + [DH + ER + KR] + ACP + KS) [15]. Es wird sich sicherlich in der Zukunft klären, ob die Implementierung der neu definierten Grenzen der PKS-Module die Verbindungsstelle des stromaufwärts liegenden ACP zu der stromabwärts befindlichen KS aufrechterhalten kann und die häufig auftretenden *Engineering*-Probleme lösen wird. Tatsächlich konnte bereits für die nicht-ribosomalen Peptidsynthetasen [16], die den PKSs sehr ähnlich sind, und die *trans*-AT-PKSs [14] gezeigt werden, dass die Aufrechterhaltung dieser Verbindungsstelle bei der Manipulation der NRP-Megasynthetasen und *trans*-AT-Megasynthasen eine kritische Rolle spielt. Die Berücksichtigung der neuen Erkenntnisse und der Einsatz von „promiskuitiven" Enzymen, wie z. B. der KirCII-AT, bieten vielversprechende Perspektiven für ein effektives PKS-*Engineering*. Unsere Arbeitsgruppe ist gerade dabei, das gewonnene Wissen zu nutzen, um das Potenzial und die allgemeine Anwendbarkeit des KirCII-ACP5-Systems als Tool für die PKS-Modifikation und die Derivatisierung von Polyketidstrukturen zu untersuchen.

## Ausblick

In den letzten drei Jahrzehnten wurde ein fundiertes Wissen bezüglich der Polyketid-Biosynthesewege und deren Schlüsselenzyme erworben. Außerdem erleichtert die Weiterentwicklung in Bereichen der Bioinformatik, Sequenzierung, synthetischen Biologie, Strukturbiologie, Biochemie, Biotechnologie und Automatisierung die *in silico*-Analysen und die experimentellen Arbeiten rund um die PKS-Systeme. Durch diese Fortschritte ist die Vision einer generell anwendbaren Hochdurchsatzstrategie zur Bereitstellung von Konstrukten für maßgeschneiderte PKSs, die der Herstellung von Polyketid-Derivaten dienen sollen, etwas realistischer geworden. Dies lässt auch neue Hoffnung für neuartige chemische Verbindungen und deren Entwicklung zu potenziellen Medikamenten aufkommen.

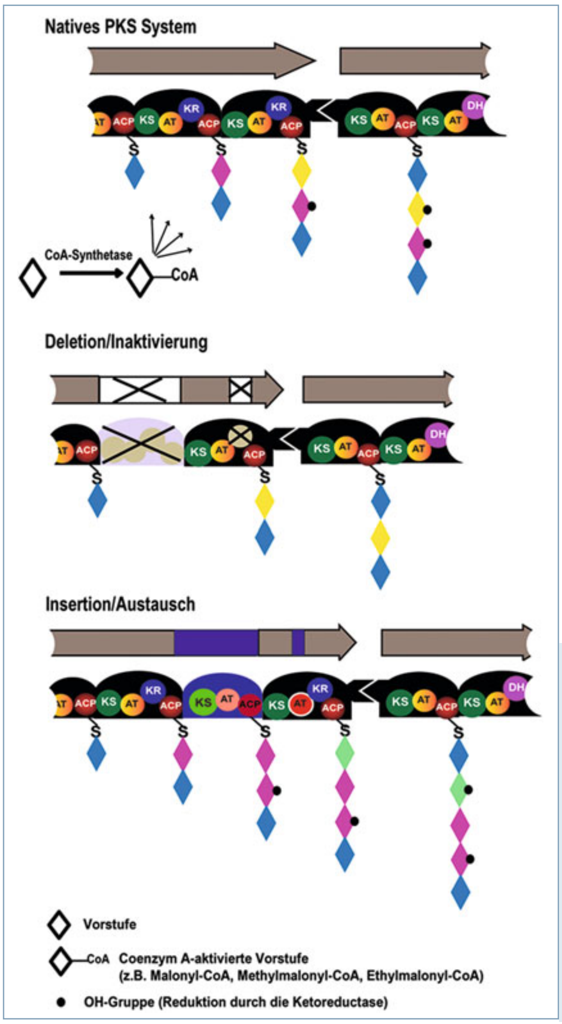

◄ **Abb. 2:** Generelle PKS-*Engineering*-Strategien. Die generellen Strategien für die Modifikation der Polyketidsynthasen (PKS) umfassen die Deletion und Inaktivierung sowie die Insertion und den Austausch von ganzen Modulen oder einzelnen Domänen der PKS. Diese Ansätze werden verwendet, um Polyketid-Derivate herzustellen (Abkürzungen siehe Abb. 1).


Funding

Open Access funding provided by Projekt DEAL.

Danksagung

Ich (E. M.-K.) danke Prof. Tilmann Weber für die erfolgreiche Zusammenarbeit. Mein besonderer Dank gilt Prof. Wolfgang Wohlleben für die Unterstützung beim Aufbau meiner Arbeitsgruppe und Förderung meiner wissenschaftlichen Karriere. Des Weiteren bedanke ich mich bei allen ehemaligen und aktuellen Mitarbeitern, die an den Arbeiten beteiligt waren und sind. Das Projekt wurde in Teilen durch das Deutsche Zentrum für Infektionsforschung (TTU09.802 und TTU09.704) die Novo Nordisk Foundation (NNF15OC0016226) und Huvepharma gefördert.


Literatur

[1] World Health Organization (2019) Antibiotic Resistance 2019. www.who.mt/news-room/fact-sheets/detail/antibiotic-resistance
[2] World Health Organization (2019) No Time to Wait: Securing the future from drug-resistant infections. Report to the Secretary-General of the United Nations, www.who.int/antimicrobial-resistance/interagency-coordination-group/final-report
[3] World Health Organization. Coronavirus disease (COVID-2019) situation reports. https://www.who.int/emergencies/diseases/novel-coronavirus-2019/situation-reports (zugegriffen am 12.3.2020)
[4] Dutta S Whicher JR Hansen DA Structure of a modular polyketide synthase Nature 2014 510 512 517 10.1038/nature13423 24965652 PMC4278352
[5] Chen H Du L Iterative polyketide biosynthesis by modular polyketide synthases in bacteria Appl Microbiol Biotechnol 2016 100 541 557 10.1007/s00253-015-7093-0 26549236 PMC4706475
[6] Hertweck C Luzhetskyy A Rebets Y Type II polyketide synthases: gaining a deeper insight into enzymatic teamwork Nat Prod Rep 2007 24 162 190 10.1039/B507395M 17268612
[7] Austin MB Noel JP The chalcone synthase superfamily of type III polyketide synthases Nat Prod Rep 2003 20 79 110 10.1039/b100917f 12636085
[8] Wolf H Zahner H Nierhaus K Kirromycin, an inhibitor of the 30S ribosomal subunits function FEBS Lett 1972 21 347 350 10.1016/0014-5793(72)80199-6 11946543
[9] Weber T Laiple KJ Pross EK Molecular analysis of the kirromycin biosynthetic gene cluster revealed beta-alanine as precursor of the pyridone moiety Chem Biol 2008 15 175 188 10.1016/j.chembiol.2007.12.009 18291322
[10] Musiol EM Weber T Discrete acyltransferases involved in polyketide biosynthesis Med Chem Comm 2012 3 871 886 10.1039/c2md20048a
[11] Musiol EM Greule A Hartner T The AT(2) domain of KirCI loads malonyl extender units to the ACPs of the kirromycin PKS Chembiochem 2013 14 1343 1352 10.1002/cbic.201300211 23828654
[12] Musiol EM Hartner T Kulik A Supramolecular templating in kirromycin biosynthesis: the acyltransferase KirCII loads ethylmalonyl-CoA extender onto a specific ACP of the trans-AT PKS Chem Biol 2011 18 438 444 10.1016/j.chembiol.2011.02.007 21513880
[13] Musiol-Kroll EM Zubeil F Schafhauser T Polyketide bioderivatization using the promiscuous acyltransferase KirCII ACS Synth Biol 2017 6 421 427 10.1021/acssynbio.6b00341 28206741 PMC5483334
[14] Vander Wood DA Keatinge-Clay AT The modules of trans-acyltransferase assembly lines redefined with a central acyl carrier protein Proteins 2018 86 664 675 10.1002/prot.25493 29524261 PMC5934314
[15] Keatinge-Clay AT Polyketide synthase modules redefined Angew Chem Int Ed Engl 2017 56 4658 4660 10.1002/anie.201701281 28322495 PMC6454894
[16] Bozhuyuk KAJ Fleischhacker F Linck A De novo design and engineering of non-ribosomal peptide synthetases Nat Chem 2018 10 275 281 10.1038/nchem.2890 29461518
